# Supplementary material for: The causal relationship between the human gut microbiota and pyogenic arthritis: a Mendelian randomization study
Source: Front Cell Infect Microbiol. 2024 Nov 26;14:1452480. doi: 10.3389/fcimb.2024.1452480 (PMC11629706; doi:10.3389/fcimb.2024.1452480)
Supplement: Supplementary file 1 [file DataSheet1.zip › mendelian test/ebi-a-GCST90027494.csv_leave_one_out.pdf]

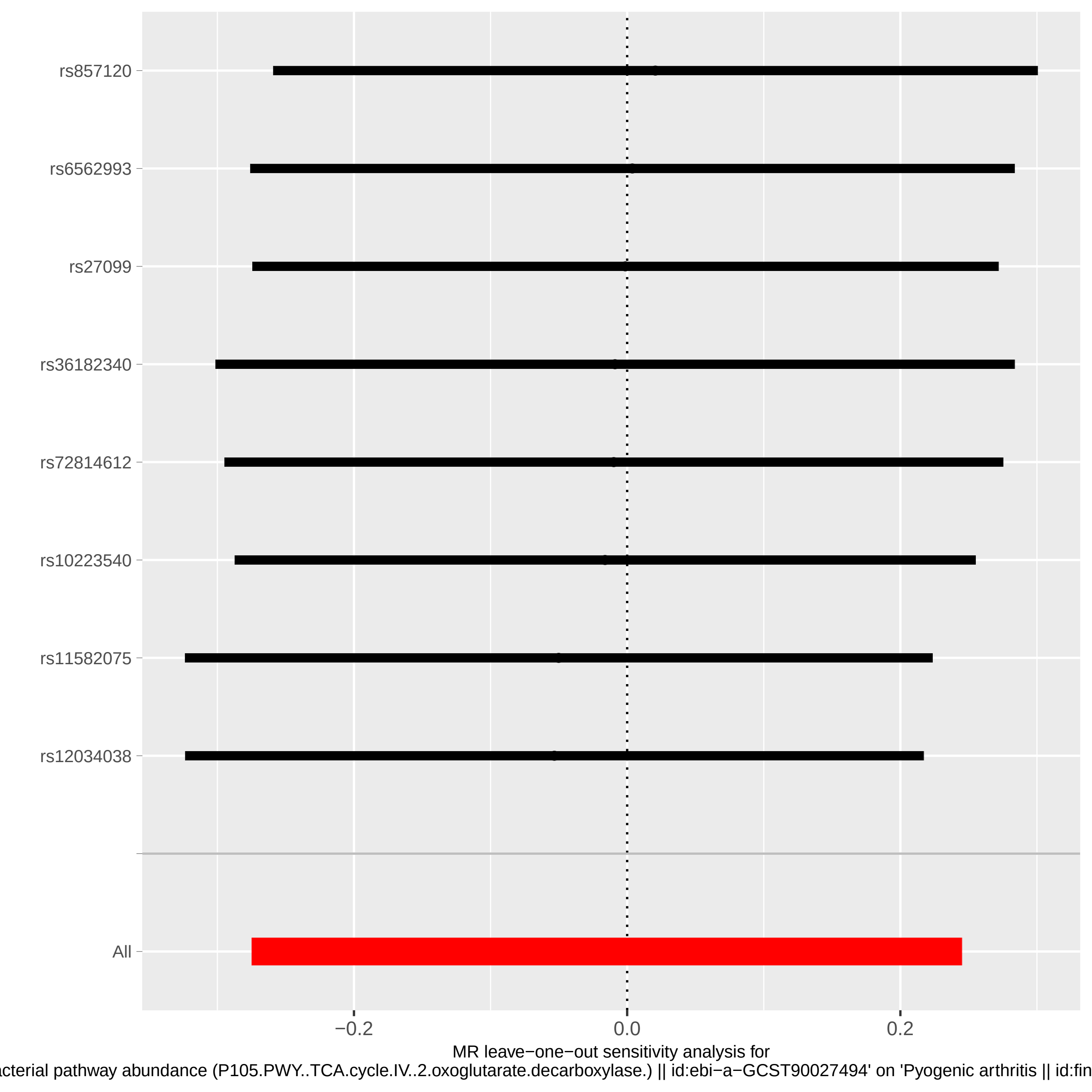
rs857120
rs6562993
rs27099
rs36182340
rs72814612
rs10223540
rs11582075
rs12034038
All
−0.2
0.0
0.2
MR leave−one−out sensitivity analysis for
acterial pathway abundance (P105.PWY..TCA.cycle.IV..2.oxoglutarate.decarboxylase.) || id:ebi−a−GCST90027494' on 'Pyogenic arthritis || id:fin
